# Increase in Prevalence of Self-Reported High Blood Cholesterol Among Adults, United States, 2019–2023

**DOI:** 10.5888/pcd23.250214

**Published:** 2026-03-05

**Authors:** Kerui Xu, Magdalena M. Pankowska, Ahlia Sekkarie, Omoye E. Imoisili, Fleetwood Loustalot

**Affiliations:** 1 Division for Heart Disease and Stroke Prevention, National Center for Chronic Disease Prevention and Health Promotion, Centers for Disease Control and Prevention, Atlanta, Georgia; 2 Alliance Group, Inc, Atlanta, Georgia

## Abstract

**Introduction:**

Elevated levels of total blood cholesterol are associated with an increased risk of atherosclerotic cardiovascular disease. Clinical guidelines and recommendations encourage regular screening and management of blood cholesterol among adults to reduce this risk. This study assessed national and state-level trends in the age-standardized prevalence of self-reported blood cholesterol screening and elevated cholesterol levels among adults by using data from the 2019–2023 Behavioral Risk Factor Surveillance System.

**Methods:**

Age-standardized prevalence of blood cholesterol screening during the preceding 5 years and high blood cholesterol among those ever screened were assessed by sex, age group, race and ethnicity, educational attainment, and state of residence. Absolute and relative changes from 2019 to 2023 were calculated, and linear and quadratic trends across survey periods were assessed by using orthogonal polynomial coefficients.

**Results:**

From 2019 to 2023, the prevalence of adults who had blood cholesterol screened during the preceding 5 years slightly decreased by 0.5%, from 86.0% to 85.6% (−0.4 percentage points, *P* = .03). Among adults who had ever been screened, the prevalence of awareness for high blood cholesterol increased by 13.7%, rising from 29.2% to 33.2% (4.0 percentage points, *P* < .001). The prevalence of screening for and awareness of high blood cholesterol varied by sociodemographic characteristics and state of residence. Approximately two-thirds of US states experienced an increase in the prevalence of self-reported high blood cholesterol.

**Conclusion:**

To improve cholesterol management, public health and clinical efforts should consider expanding resources and interventions that effectively promote early detection and raise awareness about the importance of cholesterol management.

**Summary**

**What is already on this topic?**

Elevated blood cholesterol is a major risk factor for cardiovascular disease. Blood cholesterol levels increase with age and variations exist across sociodemographic characteristics.

**What is added by this report?**

High blood cholesterol remains a serious public health challenge, with a 13.7% national increase in prevalence among adults from 2019 to 2023. The prevalence of screening and awareness for high blood cholesterol varied by sociodemographic characteristics and states. Notably, 34 states experienced an increase in the prevalence of self-reported high blood cholesterol.

**What are the implications for public health practice?**

Public health and clinical efforts should consider expanding resources and interventions that effectively promote early detection and raise awareness about the importance of cholesterol management.

## Introduction

High levels of blood cholesterol are associated with an increased risk of atherosclerotic cardiovascular disease (ASCVD) (1). Blood cholesterol levels increase with age, and differences exist across sociodemographic characteristics (2). Data from the 2017–2020 National Health and Nutrition Examination Survey (NHANES) show that approximately 34.7% of US adults had total cholesterol levels of 200 mg/dL or higher, with 10.0% at 240 mg/dL or more (1). Additionally, more than one-quarter had low-density lipoprotein cholesterol (LDL-C) levels exceeding 130 mg/dL (1). Regular screening and early intervention, including lifestyle changes and use of cholesterol-lowering medications, when necessary, can reduce the risk of ASCVD associated with high blood cholesterol. Current clinical guidelines and recommendations provide evidence-based standards for the detection, treatment, and control of high blood cholesterol (3–5).

A previous analysis using data from the 2005–2009 Behavioral Risk Factor Surveillance System (BRFSS) indicated that the prevalence of adults who had been screened for blood cholesterol and those who were told they have high blood cholesterol had both increased (6). An updated analysis of the national and state-level trends in blood cholesterol screening and awareness is essential for assessing recent changes in screening behavior. It also allows for examination of geographic and demographic variations, which can help to identify population-level differences in the prevalence of elevated blood cholesterol to guide interventions and optimize resource allocation. To achieve these objectives, we used data from the 2019–2023 BRFSS to evaluate trends in self-reported screening and awareness of high blood cholesterol among US adults.

## Methods

BRFSS is a state-based telephone survey system conducted among noninstitutionalized US adults aged 18 years or older and is the largest ongoing health survey globally, with over 400,000 participants annually (7). The median response rates for the 50 states and the District of Columbia in 2019, 2021, and 2023 were 49.4% (range, 37.3%–73.1%), 44.0% (range, 23.5%–60.5%), and 44.6% (range, 21.7%–63.1%), respectively.

Data on prevalence of screening for and awareness of high blood cholesterol were collected biennially in odd-numbered years by using the following questions: “About how long has it been since you last had your cholesterol checked?” and “Have you ever been told by a doctor, nurse, or other health professional that your cholesterol is high?” Prevalence estimates of blood cholesterol screening during the preceding 5 years and high blood cholesterol among those ever screened were assessed by sex (female and male), age group (18–44, 45–64, and ≥65 years), race and ethnicity (non-Hispanic White, non-Hispanic Black, Hispanic, non-Hispanic Asian, non-Hispanic Native Hawaiian/Pacific Islander [NH/PI], non-Hispanic American Indian/Alaska Native, and non-Hispanic Other race), educational attainment (less than high school, high school diploma, some college, and college graduate), and state of residence including the District of Columbia. As a sensitivity analysis, the prevalence estimates of blood cholesterol screening during each of the preceding 4, 3, 2, and 1 years were generated for the overall samples to examine variations in trends when focusing on more recent screening practices. All data on screening and awareness of high blood cholesterol were self-reported since BRFSS does not collect laboratory or clinical data. All percentages were age-standardized to the 2000 US Census standard population, except for age-specific estimates.

The analyses were conducted in 2024 and 2025. We calculated the absolute (percentage point) and relative (percentage) changes in prevalence from 2019 to 2023 and assessed linear and quadratic trends across survey periods using orthogonal polynomial coefficients. Significance was set at *P* < .05. All age-standardized prevalence estimates and their 95% CIs were computed using SAS-callable SUDAAN (version 11.0.4; RTI International), accounting for the BRFSS complex sampling design and weighting. To generate 95% CIs for the relative (percentage) changes, we used R statistical software (version 4.4.1; R Foundation) by sampling the normal distributions 5,000 times using the age-standardized prevalence and standard error, defining 95% CIs as the 2.5 and 97.5 percentiles of calculations of those samples. This approach was used to estimate the uncertainty of derived statistics that are not directly supported in SUDAAN, given the independent sampling design of BRFSS survey years. This activity was reviewed by the Centers for Disease Control and Prevention (CDC), deemed not research, and was conducted consistent with applicable federal law and CDC policy (8).

## Results

During 2019, 2021, and 2023, a total of 409,810, 431,639, and 425,106 respondents were interviewed, respectively. After excluding pregnant women (0.5%) and those with missing cholesterol data (6.8%–7.6%) or covariates (2.2%–2.5%), the final analytic samples for 2019, 2021, and 2023 were, respectively, 371,144 (90.6%), 385,954 (89.4%), and 383,892 (90.3%).

From 2019 to 2023, the age-standardized prevalence of adults who had been screened for high blood cholesterol during the preceding 5 years slightly decreased from 86.0% (95% CI, 85.8%–86.3%) to 85.6% (95% CI, 85.3%–85.9%), with a change of −0.4 percentage points, which represents a relative decrease of 0.5% (95% CI, −0.9% to −0.1%; *P = .*03; Table 1). In 2023, the lowest prevalence of blood cholesterol screening was found among people with less than a high school education (77.1%; 95% CI, 75.9%–78.2%) and adults aged 18 to 44 years (77.4%; 95% CI, 76.9%–77.9%). From 2019 to 2023, decreases in screening prevalence were observed in 6 states: Wyoming (−5.4%; 95% CI, −8.6% to −2.1%), Oregon (−4.8%; 95% CI, −7.0% to −2.5%), Alabama (−4.7%; 95% CI, −7.2% to −2.2%), Hawaii (−3.6%; 95% CI, −6.0% to −1.1%), Michigan (−3.1%; 95% CI, −5.0% to −1.3%), and Iowa (−2.1%; 95% CI, −4.1% to −0.1%) (*P < .*05 for all). In general, the prevalence of blood cholesterol screening (range, 79.0%–92.1%) was higher in Northeastern states and lower in Mountain West states (Figure). In terms of blood cholesterol screening within shorter time frames, we did not observe a significant change in screening prevalence from 2019 to 2023 for the 4-year, 3-year, and 2-year intervals (Table 2). However, there was a slight relative increase of 0.7% (95% CI, 0%–1.5%) in screening prevalence reported within the past year, rising from 67.0% (95% CI, 66.7%–67.4%) to 67.5% (95% CI, 67.2%–67.9%) (0.5 percentage points; *P = .*04).

**Table 1:** Age-Standardized^a^ Prevalence of Adults (Aged ≥18 Years) Who Had Been Screened for High Blood Cholesterol During the Preceding 5 Years, by Sociodemographic Characteristics and State of Residence, Behavioral Risk Factor Surveillance System, 2019–2023

Characteristic	% (95% CI)			Change from 2019 to 2023		*P* value^b^
	2019	2021	2023	Percentage point (95% CI)	Relative % (95% CI)	
**Overall, age-standardized**	86.0 (85.8 to 86.3)	84.5 (84.2 to 84.8)	85.6 (85.3 to 85.9)	−0.4 (−0.8 to −0.1)	−0.5 (−0.9 to −0.1)	.03^c^
**Overall, crude**	87.6 (87.4 to 87.9)	86.4 (86.2 to 86.7)	87.6 (87.3 to 87.8)	−0.1 (−0.4 to 0.3)	−0.1 (−0.5 to 0.3)	.68
**Sex**						
Male	83.6 (83.2 to 84.0)	81.8 (81.4 to 82.2)	82.9 (82.5 to 83.4)	−0.7 (−1.2 to −0.1)	−0.8 (−1.4 to −0.1)	.02^c^
Female	88.5 (88.2 to 88.9)	87.3 (86.9 to 87.7)	88.3 (87.9 to 88.7)	−0.3 (−0.8 to 0.3)	−0.3 (−0.9 to 0.3)	.31
**Age group, y**						
18–44	78.7 (78.2 to 79.1)	75.9 (75.4 to 76.4)	77.4 (76.9 to 77.9)	−1.3 (−2.0 to −0.6)	−1.6 (−2.5 to −0.8)	<.001^c^
45–64	92.9 (92.6 to 93.2)	92.8 (92.4 to 93.1)	93.5 (93.2 to 93.8)	0.6 (0.2 to 1.0)	0.7 (0.2 to 1.1)	.004^c^
≥65	97.1 (96.9 to 97.3)	96.9 (96.6 to 97.1)	97.4 (97.2 to 97.6)	0.3 (0.1 to 0.6)	0.3 (0.1 to 0.6)	.02^c^
**Race and ethnicity^e^**						
White, non-Hispanic	85.3 (85.0 to 85.6)	83.6 (83.3 to 84.0)	85.1 (84.8 to 85.5)	−0.2 (−0.6 to 0.3)	−0.2 (−0.7 to 0.3)	.43
Black, non-Hispanic	89.6 (88.9 to 90.3)	87.5 (86.7 to 88.2)	88.5 (87.6 to 89.2)	−1.2 (−2.3 to −0.1)	−1.3 (−2.5 to −0.1)	.04^c^
Hispanic	84.9 (84.1 to 85.6)	83.9 (83.0 to 84.6)	84.2 (83.5 to 85.0)	−0.6 (−1.7 to 0.4)	−0.7 (−1.9 to 0.5)	.23
Asian, non-Hispanic	87.7 (86.4 to 89.0)	87.3 (85.8 to 88.7)	88.3 (87.0 to 89.4)	0.5 (−1.2 to 2.3)	0.6 (−1.4 to 2.6)	.56
Native Hawaiian or Pacific Islander, non-Hispanic	85.8 (81.5 to 89.3)	85.2 (82.3 to 87.7)	81.8 (76.9 to 85.8)	−4.1 (−10.0 to 1.8)	−4.8 (−11.3 to 2.3)	.17
American Indian or Alaska Native, non-Hispanic	84.9 (83.0 to 86.7)	80.3 (78.2 to 82.3)	83.2 (81.2 to 85.1)	−1.7 (−4.4 to 1.0)	−2.0 (−5.1 to 1.2)	.22
Other Race, including multiracial, non-Hispanic	84.8 (83.4 to 86.0)	82.7 (81.3 to 84.0)	85.0 (83.7 to 86.2)	0.2 (−1.6 to 2.0)	0.2 (−1.9 to 2.4)	.83
**Education**						
Less than high school	79.5 (78.4 to 80.5)	77.5 (76.3 to 78.7)	77.1 (75.9 to 78.2)	−2.4 (−3.9 to −0.8)	−3.0 (−4.9 to −1.0)	.003^d^
High school diploma	83.4 (82.8 to 83.9)	80.7 (80.1 to 81.3)	81.7 (81.1 to 82.3)	−1.7 (−2.4 to −0.9)	−2.0 (−3.0 to −1.1)	<.001^c^
Some college	86.3 (85.9 to 86.8)	84.7 (84.2 to 85.3)	86.4 (85.9 to 87.0)	0.1 (−0.6 to 0.8)	0.1 (−0.7 to 0.9)	.75
College degree or higher	91.0 (90.7 to 91.4)	90.2 (89.8 to 90.5)	91.3 (91.0 to 91.7)	0.3 (−0.2 to 0.8)	0.3 (−0.2 to 0.8)	.21
**State of residence**						
Alabama	88.5 (87.1 to 89.7)	85.1 (83.2 to 86.8)	84.3 (82.3 to 86.1)	−4.2 (−6.5 to −1.9)	−4.7 (−7.2 to −2.2)	<.001^d^
Alaska	79.1 (76.3 to 81.7)	76.7 (74.6 to 78.6)	80.9 (79.2 to 82.5)	1.8 (−1.4 to 5.0)	2.2 (−1.8 to 6.4)	.28
Arizona	85.1 (83.5 to 86.7)	83.5 (82.3 to 84.7)	85.3 (84.0 to 86.4)	0.1 (−1.9 to 2.1)	0.1 (−2.2 to 2.5)	.90
Arkansas	83.0 (81.0 to 84.9)	83.0 (80.8 to 84.9)	82.7 (80.8 to 84.5)	−0.3 (−2.9 to 2.4)	−0.3 (−3.4 to 2.9)	.85
California	86.9 (86.1 to 87.7)	86.3 (85.1 to 87.4)	86.3 (85.3 to 87.3)	−0.6 (−1.9 to 0.7)	−0.7 (−2.1 to 0.9)	.38
Colorado	85.0 (83.8 to 86.0)	83.2 (82.2 to 84.2)	84.4 (83.2 to 85.5)	−0.5 (−2.1 to 1.0)	−0.6 (−2.4 to 1.3)	.51
Connecticut	88.3 (86.8 to 89.6)	88.4 (87.1 to 89.6)	89.9 (88.6 to 91.0)	1.6 (−0.2 to 3.4)	1.9 (−0.2 to 3.9)	.08
Delaware	87.8 (85.9 to 89.5)	87.2 (85.2 to 89.0)	86.7 (84.5 to 88.5)	−1.2 (−3.8 to 1.5)	−1.3 (−4.4 to 1.9)	.40
District of Columbia	92.3 (90.6 to 93.7)	90.0 (88.2 to 91.5)	92.1 (90.4 to 93.6)	−0.2 (−2.4 to 2.0)	−0.2 (−2.6 to 2.2)	.86
Florida^f^	86.8 (85.4 to 88.0)	—	85.5 (83.8 to 87.0)	−1.3 (−3.3 to 0.8)	−1.5 (−3.8 to 1.0)	.23
Georgia	87.6 (86.0 to 89.0)	85.3 (83.7 to 86.7)	86.5 (85.0 to 87.8)	−1.1 (−3.1 to 0.9)	−1.3 (−3.5 to 0.9)	.29
Hawaii	83.0 (81.6 to 84.2)	79.9 (78.5 to 81.3)	79.9 (78.3 to 81.5)	−3.0 (−5.1 to −1.0)	−3.6 (−6.0 to −1.1)	.004^d^
Idaho	81.0 (78.9 to 82.8)	79.6 (78.1 to 81.0)	80.5 (79.0 to 81.9)	−0.5 (−2.9 to 2.0)	−0.6 (−3.5 to 2.5)	.71
Illinois	86.4 (85.0 to 87.6)	84.5 (82.4 to 86.3)	86.7 (85.2 to 88.0)	0.3 (−1.6 to 2.2)	0.4 (−1.7 to 2.6)	.73
Indiana	83.3 (82.0 to 84.6)	81.7 (80.4 to 82.8)	84.4 (83.2 to 85.5)	1.0 (−0.7 to 2.8)	1.2 (−0.8 to 3.4)	.24
Iowa	82.9 (81.8 to 84.0)	79.5 (78.2 to 80.8)	81.2 (79.9 to 82.4)	−1.7 (−3.4 to −0.1)	−2.1 (−4.1 to −0.1)	.04^c^
Kansas	83.5 (82.4 to 84.6)	81.7 (80.8 to 82.6)	82.6 (81.3 to 83.8)	−1.0 (−2.6 to 0.7)	−1.2 (−3.1 to 0.9)	.26
Kentucky^f^	90.6 (89.2 to 91.8)	83.5 (81.8 to 85.1)	—	—	—	—
Louisiana	87.5 (85.9 to 88.9)	85.8 (84.0 to 87.4)	86.7 (85.1 to 88.2)	−0.8 (−2.9 to 1.4)	−0.9 (−3.3 to 1.6)	.49
Maine	85.8 (84.1 to 87.4)	64.8 (63.1 to 66.4)	84.0 (82.4 to 85.4)	−1.9 (−4.1 to 0.3)	−2.2 (−4.6 to 0.4)	.10
Maryland	90.4 (89.5 to 91.3)	88.0 (87.0 to 89.0)	89.8 (88.9 to 90.7)	−0.6 (−1.9 to 0.7)	−0.6 (−2.1 to 0.8)	.37
Massachusetts	89.5 (88.3 to 90.6)	87.8 (86.6 to 89.0)	90.0 (88.9 to 91.1)	0.5 (−1.1 to 2.1)	0.6 (−1.2 to 2.4)	.51
Michigan	89.0 (87.9 to 90.0)	85.0 (83.8 to 86.2)	86.2 (84.9 to 87.4)	−2.8 (−4.5 to −1.1)	−3.1 (−5.0 to −1.3)	<.001^c^
Minnesota	84.2 (83.3 to 85.1)	82.2 (81.2 to 83.0)	84.5 (83.4 to 85.5)	0.3 (−1.1 to 1.6)	0.3 (−1.3 to 1.9)	.69
Mississippi	86.5 (84.9 to 88.0)	82.8 (80.6 to 84.7)	85.4 (83.5 to 87.2)	−1.1 (−3.5 to 1.3)	−1.3 (−4.1 to 1.5)	.37
Missouri	83.9 (82.4 to 85.4)	82.7 (81.3 to 83.9)	82.1 (80.5 to 83.6)	−1.8 (−4.0 to 0.4)	−2.2 (−4.6 to 0.4)	.10
Montana	80.1 (78.6 to 81.5)	77.5 (75.8 to 79.1)	79.2 (77.6 to 80.7)	−1.0 (−3.1 to 1.2)	−1.2 (−3.8 to 1.5)	.38
Nebraska	81.8 (80.7 to 82.8)	80.2 (79.1 to 81.2)	83.4 (82.3 to 84.4)	1.6 (0.1 to 3.1)	1.9 (0.1 to 3.7)	.04^c^
Nevada	84.4 (82.2 to 86.3)	83.0 (80.6 to 85.2)	84.1 (81.1 to 86.7)	−0.3 (−3.8 to 3.1)	−0.4 (−4.5 to 3.8)	.85
New Hampshire	84.3 (82.2 to 86.2)	84.8 (83.0 to 86.5)	87.2 (85.2 to 88.9)	2.9 (0.2 to 5.6)	3.4 (0.4 to 6.8)	.04^d^
New Jersey^f^	—	89.1 (87.9 to 90.1)	89.5 (88.3 to 90.5)	—	—	—
New Mexico	80.7 (78.9 to 82.4)	77.7 (76.0 to 79.3)	82.4 (79.8 to 84.8)	1.7 (−1.3 to 4.7)	2.1 (−1.6 to 6.0)	.27
New York	88.4 (87.4 to 89.4)	87.8 (87.1 to 88.5)	88.7 (87.8 to 89.5)	0.2 (−1.1 to 1.6)	0.3 (−1.2 to 1.8)	.72
North Carolina	86.8 (85.3 to 88.2)	85.8 (84.2 to 87.3)	88.0 (86.3 to 89.6)	1.2 (−1.0 to 3.4)	1.4 (−1.1 to 4.0)	.30
North Dakota	79.9 (77.9 to 81.8)	77.8 (76.1 to 79.5)	82.3 (80.7 to 83.8)	2.3 (−0.1 to 4.8)	2.9 (−0.1 to 6.2)	.07
Ohio	82.9 (81.6 to 84.2)	82.2 (81.0 to 83.3)	83.9 (82.6 to 85.1)	1.0 (−0.8 to 2.8)	1.2 (−1.0 to 3.4)	.29
Oklahoma	84.1 (82.6 to 85.5)	79.9 (78.1 to 81.5)	82.5 (81.1 to 83.9)	−1.6 (−3.6 to 0.4)	−1.9 (−4.2 to 0.5)	.12
Oregon	86.7 (85.5 to 87.9)	81.3 (79.8 to 82.7)	82.5 (81.0 to 84.0)	−4.2 (−6.1 to −2.3)	−4.8 (−7.0 to −2.5)	<.001^c^
Pennsylvania^f^	85.5 (84.0 to 86.9)	84.5 (83.0 to 85.9)	—	—	—	—
Rhode Island	90.7 (89.1 to 92.1)	89.0 (87.3 to 90.4)	89.1 (87.4 to 90.6)	−1.7 (−3.9 to 0.5)	−1.8 (−4.2 to 0.6)	.14
South Carolina	83.5 (82.0 to 84.9)	84.0 (82.5 to 85.4)	84.7 (83.3 to 86.1)	1.2 (−0.8 to 3.2)	1.5 (−0.9 to 4.0)	.24
South Dakota	77.5 (75.0 to 79.9)	81.4 (78.6 to 83.8)	86.1 (83.7 to 88.1)	8.5 (5.3 to 11.8)	11.0 (6.4 to 15.7)	<.001^d^
Tennessee	87.9 (86.4 to 89.3)	86.7 (85.1 to 88.2)	87.1 (85.6 to 88.5)	−0.8 (−2.8 to 1.2)	−0.9 (−3.2 to 1.4)	.44
Texas	85.2 (83.8 to 86.5)	83.9 (82.4 to 85.3)	83.6 (82.0 to 85.0)	−1.6 (−3.6 to 0.4)	−1.9 (−4.2 to 0.5)	.11
Utah	81.9 (80.9 to 82.9)	80.8 (79.7 to 81.9)	81.4 (80.3 to 82.5)	−0.5 (−2.0 to 1.0)	−0.6 (−2.4 to 1.2)	.51
Vermont	79.6 (77.3 to 81.7)	80.5 (78.5 to 82.4)	83.6 (81.9 to 85.2)	4.0 (1.3 to 6.8)	5.1 (1.5 to 8.8)	.004^d^
Virginia	87.8 (86.6 to 88.9)	86.3 (85.0 to 87.5)	87.1 (85.4 to 88.5)	−0.7 (−2.6 to 1.2)	−0.8 (−3.0 to 1.4)	.46
Washington	82.8 (81.8 to 83.8)	81.8 (80.7 to 82.8)	83.1 (82.3 to 83.8)	0.2 (−1.0 to 1.5)	0.3 (−1.2 to 1.9)	.70
West Virginia	85.0 (83.0 to 86.7)	84.8 (83.2 to 86.2)	85.3 (83.3 to 87.0)	0.3 (−2.3 to 2.9)	0.4 (−2.6 to 3.5)	.82
Wisconsin	82.0 (80.2 to 83.7)	81.3 (79.5 to 83.0)	83.6 (82.4 to 84.8)	1.6 (−0.5 to 3.8)	2.0 (−0.6 to 4.6)	.14
Wyoming	83.5 (81.5 to 85.3)	77.1 (74.6 to 79.3)	79.0 (76.9 to 80.9)	−4.5 (−7.3 to −1.7)	−5.4 (−8.6 to −2.1)	.001^c^

Abbreviation: — , not applicable.

a Age-standardized to the 2000 US. Census Bureau standard population, for all characteristics except age group.

b Trends across survey periods were assessed using orthogonal polynomial coefficients; *P *value was reported based on linear trend analysis.

c Significant at *P* < .05 for linear trend across survey periods.

d Significant at *P* < .05 for both linear and quadratic trends across survey periods.

e People identified as Hispanic might be of any race. People identified as White, Black, Asian, Native Hawaiian or Pacific Islander, American Indian or Alaska Native, or Other race are all non-Hispanic.

f New Jersey (2019), Florida (2021), Kentucky (2023), and Pennsylvania (2023) were unable to collect sufficient data to meet the minimum requirements for inclusion in the BRFSS public-use data set.

**Figure:**
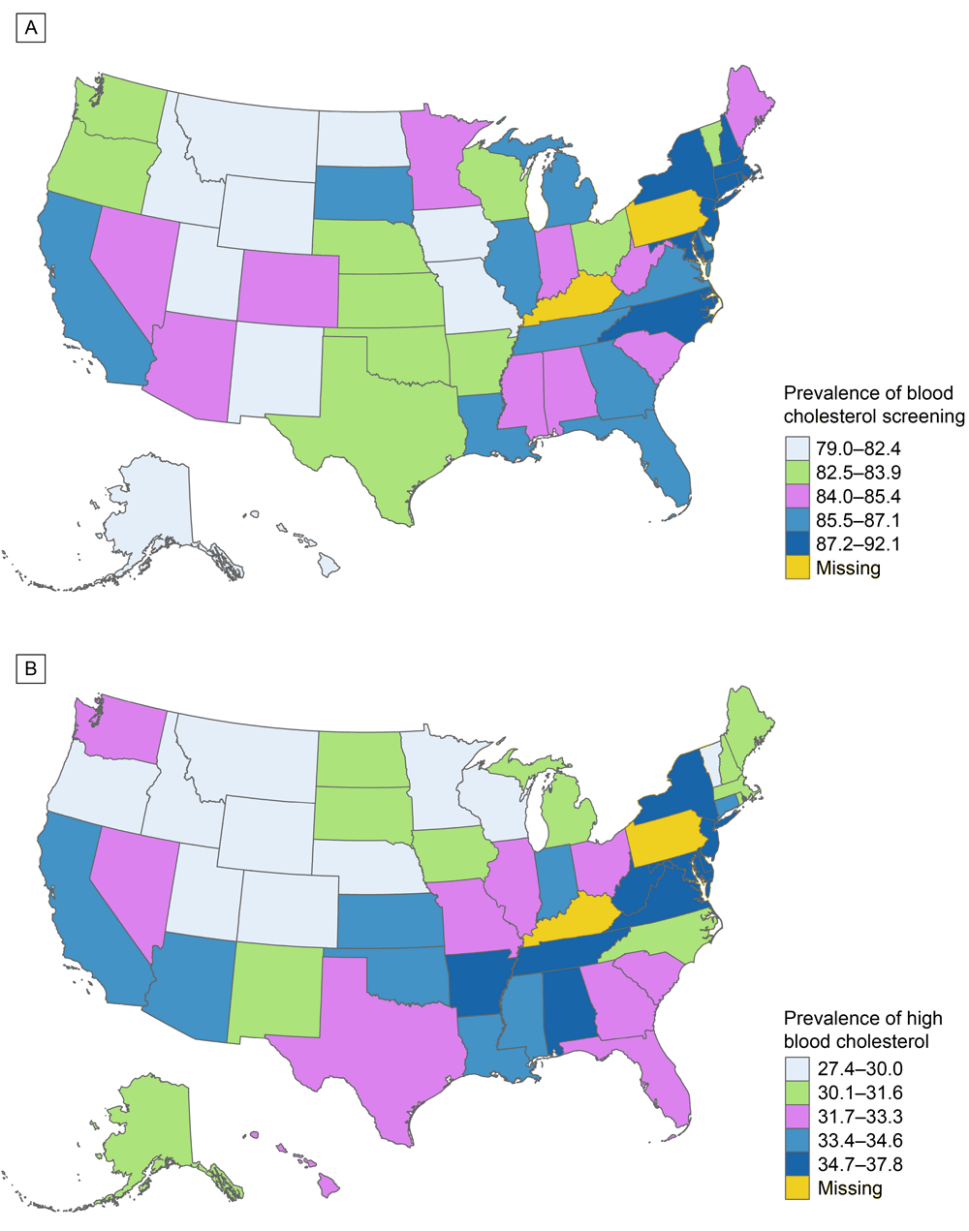
Age-standardized prevalence of self-reported blood cholesterol screening and high blood cholesterol among US adults (aged ≥18 y), Behavioral Risk Factor Surveillance System, 2023. Map A shows the prevalence of US adults who had been screened for high blood cholesterol in the preceding 5 years, and Map B shows the prevalence of adults who had ever been screened for high blood cholesterol and were told by a health care provider that they have high blood cholesterol. Data are categorized by state of residence. Kentucky and Pennsylvania were unable to collect sufficient data in 2023 to meet the minimum requirements for inclusion in the BRFSS public-use data set. Abbreviation: — , data not available.

**Table:** 

State of residence	Map A, quintile range, %	Map B, quintile range, %
Alabama	84.0–85.4	34.7–37.8
Alaska	79.0–82.4	30.1–31.6
Arizona	84.0–85.4	33.4–34.6
Arkansas	82.5–83.9	34.7–37.8
California	85.5–87.1	33.4–34.6
Colorado	84.0–85.4	27.4–30.0
Connecticut	87.2–92.1	33.4–34.6
Delaware	85.5–87.1	34.7–37.8
District of Columbia	87.2–92.1	33.4–34.6
Florida	85.5–87.1	31.7–33.3
Georgia	85.5–87.1	31.7–33.3
Hawaii	79.0–82.4	31.7–33.3
Idaho	79.0–82.4	27.4–30.0
Illinois	85.5–87.1	31.7–33.3
Indiana	84.0–85.4	33.4–34.6
Iowa	79.0–82.4	30.1–31.6
Kansas	82.5–83.9	33.4–34.6
Kentucky	—	—
Louisiana	85.5–87.1	33.4–34.6
Maine	84.0–85.4	30.1–31.6
Maryland	87.2–92.1	34.7–37.8
Massachusetts	87.2–92.1	30.1–31.6
Michigan	85.5–87.1	30.1–31.6
Minnesota	84.0–85.4	27.4–30.0
Mississippi	84.0–85.4	33.4–34.6
Missouri	79.0–82.4	31.7–33.3
Montana	79.0–82.4	27.4–30.0
Nebraska	82.5–83.9	27.4–30.0
Nevada	84.0–85.4	31.7–33.3
New Hampshire	87.2–92.1	30.1–31.6
New Jersey	87.2–92.1	34.7–37.8
New Mexico	79.0–82.4	30.1–31.6
New York	87.2–92.1	34.7–37.8
North Carolina	87.2–92.1	30.1–31.6
North Dakota	79.0–82.4	30.1–31.6
Ohio	82.5–83.9	31.7–33.3
Oklahoma	82.5–83.9	33.4–34.6
Oregon	82.5–83.9	27.4–30.0
Pennsylvania	—	—
Rhode Island	87.2–92.1	30.1–31.6
South Carolina	84.0–85.4	31.7–33.3
South Dakota	85.5–87.1	30.1–31.6
Tennessee	85.5–87.1	34.7–37.8
Texas	82.5–83.9	31.7–33.3
Utah	79.0–82.4	27.4–30.0
Vermont	82.5–83.9	27.4–30.0
Virginia	85.5–87.1	34.7–37.8
Washington	82.5–83.9	31.7–33.3
West Virginia	84.0–85.4	34.7–37.8
Wisconsin	82.5–83.9	27.4–30.0
Wyoming	79.0–82.4	27.4–30.0

**Table 2:** Age-Standardized^a^ Prevalence of Adults (≥18 Years) Who Had Been Screened For High Blood Cholesterol During the Preceding 5, 4, 3, 2, and 1 Years, Behavioral Risk Factor Surveillance System, 2019–2023

Screening time	% (95% CI)			Change from 2019 to 2023		*P* value^b^
	2019	2021	2023	Percentage point (95% CI)	Relative % (95% CI)	
Overall, screened in past 5 years	86.0 (85.8 to 86.3)	84.5 (84.2 to 84.8)	85.6 (85.3 to 85.9)	−0.4 (−0.8 to −0.1)	−0.5 (−0.9 to −0.1)	.03^c^

Overall, screened in past 4 years	84.5 (84.2 to 84.7)	82.9 (82.6 to 83.2)	84.3 (84.0 to 84.6)	−0.2 (−0.6 to 0.2)	−0.2 (−0.7 to 0.3)	.34

Overall, screened in past 3 years	82.8 (82.5 to 83.1)	81.2 (80.9 to 81.5)	82.5 (82.2 to 82.8)	−0.3 (−0.7 to 0.2)	−0.3 (−0.8 to 0.2)	.22

Overall, screened in past 2 years	78.7 (78.4 to 79.0)	76.3 (76.0 to 76.7)	78.7 (78.4 to 79.0)	−0.03 (−0.5 to 0.4)	−0.03 (−0.6 to 0.5)	.91

Overall, screened in past 1 year	67.0 (66.7 to 67.4)	62.6 (62.2 to 62.9)	67.5 (67.2 to 67.9)	0.5 (0.0 to 1.0)	0.7 (0.0 to 1.5)	.04^c^

a Age-standardized to the 2000 U.S. Census Bureau standard population.

b Trends across survey periods were assessed using orthogonal polynomial coefficients.

c Significant at *P* < .05 for both linear and quadratic trends across survey periods. *P* value reported based on linear trend analysis.

From 2019 to 2023, among adults who had ever been screened, the age-standardized prevalence of those reporting awareness of high blood cholesterol increased by 13.7% (95% CI, 12.0%–15.4%), rising from 29.2% (95% CI, 28.9%–29.5%) to 33.2% (95% CI, 32.8%–33.5%), representing a change of 4.0 percentage points (*P < .*001; Table 3). Similar increases were observed across sociodemographic subgroups. By sex, we identified a relative increase of 12.7% (95% CI, 10.4%–15.1%; *P < .*001) in men, rising from 30.7% (95% CI, 30.3%–31.2%) to 34.6% (95% CI, 34.1%–35.1%). In women, there was a 14.8% increase (95% CI, 12.3%–17.3%; *P < .*001), from 27.7% (95% CI, 27.3%–28.1%) to 31.8% (95% CI, 31.3%–32.3%). The greatest relative increases were seen among non-Hispanic NH/PI adults (42.9%; 95% CI, 9.9%–88.8%; *P = .*009), adults aged 18 to 44 years (28.3%; 95% CI, 23.5%–33.5%; *P < .*001), and non-Hispanic Asian adults (27.6%; 95% CI, 16.6%–39.5%; *P < .*001). In 2023, the highest prevalence of self-reported high blood cholesterol was observed among older adults aged ≥65 years (55.1%; 95% CI, 54.5%–55.7%). From 2019 to 2023, 34 states experienced a significant increase in the prevalence of awareness for high blood cholesterol, with notable increases in Delaware (26.9%; 95% CI, 14.8%–40.8%), South Dakota (26.3%; 95% CI, 10.5%–44.7%), and North Dakota (26.0%; 95% CI, 16.4%–36.5%) (*P < .*05 for all). The prevalence of self-reported high blood cholesterol (range, 27.4%–37.8%) was, in general, higher in Appalachian and Southern states and lower in Mountain West states (Figure).

**Table 3:** Age-Standardized^a^ Prevalence of Adults (Aged ≥18 Years) Who Were Told by a Health Care Provider They Have High Blood Cholesterol Among Those Ever Screened for Blood Cholesterol, by Sociodemographic Characteristics and State of Residence, Behavioral Risk Factor Surveillance System, 2019–2023

Characteristic	% (95% CI)			Change from 2019 to 2023		*P* value^b^
	2019	2021	2023	Percentage point (95% CI)	Relative % (95% CI)	
**Overall, age-standardized**	29.2 (28.9 to 29.5)	31.5 (31.1 to 31.8)	33.2 (32.8 to 33.5)	4.0 (3.5 to 4.5)	13.7 (12.0 to 15.4)	<.001^c^
**Overall, crude**	33.2 (32.8 to 33.5)	35.7 (35.3 to 36.0)	37.6 (37.2 to 37.9)	4.4 (3.9 to 4.9)	13.3 (11.8 to 14.9)	<.001^c^
**Sex**						
Male	30.7 (30.3 to 31.2)	33.0 (32.5 to 33.5)	34.6 (34.1 to 35.1)	3.9 (3.2 to 4.6)	12.7 (10.4 to 15.1)	<.001^c^
Female	27.7 (27.3 to 28.1)	30.0 (29.5 to 30.5)	31.8 (31.3 to 32.3)	4.1 (3.5 to 4.7)	14.8 (12.3 to 17.3)	<.001^c^
**Age group, y**						
18–44	16.0 (15.5 to 16.4)	18.6 (18.1 to 19.1)	20.5 (19.9 to 21.0)	4.5 (3.8 to 5.2)	28.3 (23.5 to 33.5)	<.001^c^
45–64	40.1 (39.5 to 40.6)	41.7 (41.1 to 42.3)	43.2 (42.6 to 43.8)	3.2 (2.3 to 4.0)	7.9 (5.7 to 10.0)	<.001^c^
≥65	51.3 (50.8 to 51.9)	53.6 (53.0 to 54.3)	55.1 (54.5 to 55.7)	3.8 (3.0 to 4.6)	7.4 (5.8 to 9.2)	<.001^c^
**Race/ethnicity^d^**						
White, non-Hispanic	29.3 (28.9 to 29.6)	31.3 (31.0 to 31.7)	32.8 (32.5 to 33.2)	3.6 (3.1 to 4.1)	12.3 (10.4 to 14.1)	<.001^c^
Black, non-Hispanic	28.0 (27.1 to 28.9)	29.7 (28.8 to 30.6)	31.1 (30.1 to 32.1)	3.1 (1.8 to 4.4)	11.1 (6.2 to 16.1)	<.001^c^
Hispanic	29.2 (28.2 to 30.2)	31.8 (30.7 to 33.0)	33.7 (32.6 to 34.8)	4.5 (3.0 to 6.0)	15.4 (10.0 to 20.9)	<.001^c^
Asian, non-Hispanic	28.4 (26.5 to 30.4)	32.3 (30.0 to 34.7)	36.3 (34.2 to 38.5)	7.9 (5.0 to 10.7)	27.6 (16.6 to 39.5)	<.001^c^
Native Hawaiian or Pacific Islander, non-Hispanic	23.3 (19.0 to 28.2)	29.9 (24.8 to 35.6)	33.2 (27.7 to 39.3)	10.0 (2.5 to 17.4)	42.9 (9.9 to 88.8)	.009^c^
American Indian or Alaska Native, non-Hispanic	30.2 (27.8 to 32.8)	27.5 (25.4 to 29.7)	33.0 (29.8 to 36.3)	2.7 (−1.3 to 6.8)	9.1 (−4.3 to 24.1)	.18
Other Race, including multiracial, non-Hispanic	29.2 (27.5 to 31.0)	32.0 (30.1 to 33.9)	31.8 (30.1 to 33.6)	2.6 (0.1 to 5.1)	9.0 (0.4 to 17.9)	.04^c^
**Education**						
Less than high school	34.3 (33.0 to 35.5)	34.3 (33.0 to 35.7)	36.1 (34.7 to 37.5)	1.8 (−0.1 to 3.7)	5.3 (−0.3 to 11.0)	.06
High school diploma	28.5 (27.9 to 29.0)	31.1 (30.5 to 31.8)	32.5 (31.8 to 33.3)	4.1 (3.2 to 5.0)	14.3 (11.0 to 18.0)	<.001^c^
Some college	29.0 (28.4 to 29.5)	31.2 (30.5 to 31.8)	32.9 (32.2 to 33.5)	3.9 (3.0 to 4.8)	13.5 (10.3 to 16.7)	<.001^c^
College degree or higher	28.2 (27.7 to 28.7)	31.0 (30.5 to 31.5)	33.2 (32.7 to 33.7)	5.0 (4.3 to 5.7)	17.7 (15.0 to 20.5)	<.001^c^
**State of residence**						
Alabama	32.3 (30.8 to 33.9)	34.2 (32.2 to 36.2)	37.0 (34.9 to 39.1)	4.7 (2.0 to 7.3)	14.4 (6.3 to 23.4)	<.001^c^
Alaska	25.8 (23.5 to 28.1)	28.3 (26.4 to 30.4)	30.1 (28.3 to 31.8)	4.3 (1.4 to 7.2)	16.7 (4.9 to 30.2)	.004^c^
Arizona	29.8 (28.1 to 31.6)	31.0 (29.7 to 32.4)	34.4 (32.9 to 35.9)	4.5 (2.2 to 6.9)	15.2 (6.8 to 24.1)	<.001^c^
Arkansas	32.2 (30.4 to 34.1)	31.9 (29.9 to 33.9)	34.7 (32.8 to 36.6)	2.4 (−0.2 to 5.1)	7.6 (−0.7 to 16.6)	.07
California	27.6 (26.5 to 28.7)	31.2 (29.7 to 32.8)	34.1 (32.7 to 35.6)	6.6 (4.8 to 8.4)	23.8 (16.9 to 30.9)	<.001^c^
Colorado	26.9 (25.8 to 28.1)	28.9 (27.8 to 30.0)	29.0 (27.8 to 30.3)	2.2 (0.4 to 3.9)	8.0 (1.5 to 14.5)	.01^c^
Connecticut	30.5 (29.0 to 32.0)	31.1 (29.5 to 32.7)	34.1 (32.5 to 35.7)	3.6 (1.4 to 5.8)	11.9 (4.5 to 20.0)	.001^c^
Delaware	29.8 (27.7 to 31.9)	32.3 (30.1 to 34.7)	37.8 (35.2 to 40.4)	8.0 (4.6 to 11.4)	26.9 (14.8 to 40.8)	<.001^c^
District of Columbia	28.3 (26.2 to 30.4)	30.6 (28.5 to 32.7)	33.5 (31.2 to 35.8)	5.2 (2.1 to 8.3)	18.4 (7.0 to 31.2)	.001^c^
Florida^e^	28.2 (26.6 to 29.8)	—	33.2 (31.3 to 35.2)	5.1 (2.6 to 7.6)	18.0 (9.1 to 27.9)	<.001^c^
Georgia	29.1 (27.4 to 30.8)	33.1 (31.5 to 34.8)	33.3 (31.7 to 35.0)	4.2 (1.9 to 6.6)	14.6 (5.8 to 24.0)	<.001^c^
Hawaii	26.1 (24.7 to 27.5)	29.9 (28.3 to 31.6)	31.9 (30.2 to 33.7)	5.8 (3.6 to 8.1)	22.4 (13.7 to 32.3)	<.001^c^
Idaho	27.3 (25.4 to 29.3)	28.7 (27.2 to 30.2)	30.0 (28.3 to 31.8)	2.7 (0.0 to 5.3)	9.8 (0.3 to 20.2)	.047^c^
Illinois	27.8 (26.5 to 29.2)	28.0 (25.9 to 30.1)	32.3 (30.6 to 34.2)	4.5 (2.3 to 6.8)	16.3 (7.8 to 25.2)	<.001^c^
Indiana	29.1 (27.9 to 30.4)	30.8 (29.6 to 32.0)	34.1 (32.9 to 35.4)	5.0 (3.2 to 6.8)	17.1 (10.7 to 24.1)	<.001^c^
Iowa	28.1 (27.0 to 29.3)	29.3 (28.0 to 30.6)	31.3 (29.9 to 32.7)	3.1 (1.4 to 4.9)	11.2 (4.7 to 17.6)	<.001^c^
Kansas	30.4 (29.2 to 31.6)	33.7 (32.7 to 34.7)	33.4 (32.0 to 34.8)	3.0 (1.2 to 4.8)	9.9 (3.8 to 16.4)	.001^f^
Kentucky^e^	33.0 (31.3 to 34.8)	33.0 (31.1 to 34.9)	—	—	—	—
Louisiana	33.6 (31.8 to 35.5)	33.7 (31.8 to 35.7)	34.6 (32.8 to 36.5)	1.0 (−1.6 to 3.6)	3.0 (−4.6 to 11.1)	.44
Maine	28.1 (26.6 to 29.7)	28.1 (26.6 to 29.6)	30.8 (29.4 to 32.2)	2.7 (0.5 to 4.8)	9.5 (1.8 to 17.5)	.01^c^
Maryland	31.7 (30.5 to 32.9)	33.5 (32.4 to 34.7)	35.6 (34.3 to 36.9)	3.9 (2.2 to 5.7)	12.4 (6.7 to 18.3)	<.001^c^
Massachusetts	26.9 (25.5 to 28.2)	31.1 (29.6 to 32.5)	31.6 (30.1 to 33.1)	4.7 (2.7 to 6.8)	17.6 (9.7 to 26.0)	<.001^f^
Michigan	29.8 (28.6 to 31.0)	31.9 (30.6 to 33.3)	30.7 (29.3 to 32.1)	0.9 (−0.9 to 2.7)	3.0 (−3.0 to 9.6)	.34
Minnesota	25.7 (24.9 to 26.6)	26.8 (25.9 to 27.7)	29.2 (28.0 to 30.4)	3.4 (2.0 to 4.9)	13.4 (7.5 to 19.4)	<.001^c^
Mississippi	31.8 (30.1 to 33.5)	33.0 (31.0 to 35.0)	34.4 (32.2 to 36.7)	2.6 (−0.2 to 5.5)	8.2 (−0.6 to 17.9)	.07
Missouri	30.4 (28.8 to 32.1)	31.0 (29.5 to 32.4)	32.7 (31.1 to 34.4)	2.3 (0.0 to 4.7)	7.6 (−0.1 to 15.7)	.05
Montana	24.8 (23.5 to 26.2)	25.2 (23.7 to 26.9)	28.1 (26.5 to 29.7)	3.3 (1.2 to 5.4)	13.2 (4.4 to 22.5)	.002^c^
Nebraska	26.7 (25.7 to 27.7)	29.8 (28.7 to 31.0)	29.8 (28.7 to 31.0)	3.1 (1.6 to 4.7)	11.7 (5.6 to 17.6)	<.001^f^
Nevada	30.7 (28.2 to 33.3)	33.2 (30.2 to 36.3)	33.3 (30.2 to 36.5)	2.6 (−1.5 to 6.7)	8.5 (−4.5 to 22.9)	.21
New Hampshire	28.3 (26.4 to 30.3)	27.6 (25.8 to 29.4)	30.1 (28.2 to 32.1)	1.8 (−0.9 to 4.6)	6.5 (−2.9 to 16.7)	.19
New Jersey^e^	—	33.4 (31.9 to 35.0)	35.5 (34.0 to 37.1)	—	—	—
New Mexico	26.7 (25.0 to 28.4)	30.8 (29.0 to 32.6)	30.9 (28.1 to 33.8)	4.2 (1.0 to 7.5)	15.8 (3.7 to 28.8)	.01^c^
New York	29.0 (27.9 to 30.2)	32.5 (31.5 to 33.4)	34.7 (33.6 to 35.9)	5.7 (4.1 to 7.3)	19.7 (13.6 to 26.0)	<.001^c^
North Carolina	30.5 (28.8 to 32.2)	31.7 (30.0 to 33.4)	31.6 (29.5 to 33.8)	1.1 (−1.6 to 3.8)	3.7 (−4.7 to 13.3)	.42
North Dakota	24.5 (23.1 to 26.0)	29.3 (27.6 to 31.1)	30.9 (29.3 to 32.6)	6.4 (4.2 to 8.6)	26.0 (16.4 to 36.5)	<.001^c^
Ohio	28.2 (26.9 to 29.5)	30.5 (29.3 to 31.7)	32.0 (30.7 to 33.3)	3.8 (2.0 to 5.7)	13.6 (6.9 to 20.5)	<.001^c^
Oklahoma	31.8 (30.3 to 33.4)	32.5 (30.7 to 34.3)	33.7 (32.2 to 35.2)	1.9 (−0.3 to 4.0)	5.8 (−0.9 to 13.1)	.09
Oregon	26.4 (24.9 to 27.8)	27.9 (26.3 to 29.5)	29.3 (27.7 to 30.9)	3.0 (0.8 to 5.1)	11.2 (3.1 to 20.2)	.007^c^
Pennsylvania^e^	28.5 (27.0 to 30.1)	29.1 (27.6 to 30.7)	—	—	—	—
Rhode Island	28.7 (26.9 to 30.6)	29.7 (28.0 to 31.4)	30.1 (28.2 to 32.1)	1.4 (−1.3 to 4.1)	4.9 (−4.5 to 15.1)	.31
South Carolina	31.2 (29.7 to 32.8)	32.1 (30.6 to 33.5)	33.2 (31.8 to 34.7)	2.0 (−0.1 to 4.1)	6.5 (−0.2 to 13.6)	.06
South Dakota	24.4 (22.5 to 26.4)	31.1 (28.0 to 34.3)	30.8 (27.6 to 34.2)	6.4 (2.6 to 10.2)	26.3 (10.5 to 44.7)	.001^c^
Tennessee	31.7 (30.0 to 33.4)	33.7 (31.8 to 35.6)	36.2 (34.3 to 38.2)	4.5 (1.9 to 7.1)	14.2 (6.2 to 23.2)	<.001^c^
Texas	32.0 (30.3 to 33.7)	33.2 (31.5 to 35.0)	33.3 (31.4 to 35.2)	1.4 (−1.2 to 3.9)	4.3 (−3.6 to 12.8)	.30
Utah	28.0 (26.9 to 29.1)	29.7 (28.5 to 30.9)	29.3 (28.2 to 30.5)	1.4 (−0.2 to 3.0)	4.9 (−0.8 to 11.0)	.10
Vermont	24.7 (23.1 to 26.4)	26.6 (24.9 to 28.5)	27.4 (25.9 to 29.1)	2.7 (0.4 to 5.0)	10.9 (1.5 to 21.2)	.02^c^
Virginia	29.2 (27.8 to 30.5)	34.3 (32.9 to 35.8)	35.2 (33.3 to 37.1)	6.0 (3.7 to 8.4)	20.7 (12.4 to 29.4)	<.001^f^
Washington	27.9 (26.9 to 29.0)	29.5 (28.4 to 30.6)	32.0 (31.2 to 32.8)	4.1 (2.8 to 5.4)	14.8 (9.7 to 20.0)	<.001^c^
West Virginia	33.6 (31.8 to 35.4)	34.3 (32.7 to 35.9)	37.4 (35.4 to 39.4)	3.8 (1.1 to 6.5)	11.4 (3.6 to 20.2)	.005^c^
Wisconsin	28.7 (26.9 to 30.4)	30.6 (28.9 to 32.5)	30.0 (28.8 to 31.3)	1.4 (−0.8 to 3.5)	4.7 (−2.7 to 12.9)	.22
Wyoming	23.9 (22.1 to 25.8)	27.0 (24.8 to 29.4)	28.8 (26.8 to 30.7)	4.9 (2.1 to 7.6)	20.3 (8.7 to 33.6)	<.001^c^

Abbreviation: — , not applicable.

a Age-standardized to the 2000 US Census Bureau standard population for all characteristics except age group.

b Trends across survey periods were assessed using orthogonal polynomial coefficients; *P *value was reported based on linear trend analysis.

c Significant at *P* < .05 for linear trend across survey periods.

d People identified as Hispanic might be of any race. People identified as White, Black, Asian, Native Hawaiian or Pacific Islander, American Indian or Alaska Native, or Other race are all non-Hispanic.

e New Jersey (2019), Florida (2021), Kentucky (2023), and Pennsylvania (2023) were unable to collect sufficient data to meet the minimum requirements for inclusion in the BRFSS public-use data set.

f Significant at *P* < .05 for both linefar and quadratic trends across survey periods.

## Discussion

This study is the first in more than a decade to report on the US national and state-level prevalence of screening and awareness for elevated blood cholesterol levels. From 2019 to 2023, a slight decrease of 0.5% was found in the age-standardized prevalence of adults who had been screened during the preceding 5 years. Concurrently, the age-standardized prevalence of adults who were told by a health care provider they have high blood cholesterol increased by 13.7%. Notably, approximately two-thirds of US states experienced an increased prevalence in self-reported high blood cholesterol.

Current guidelines recommend regular blood cholesterol screening and ASCVD risk assessment for most healthy adults aged 20 years or older every 4 to 6 years (3,4). Our findings differ from an earlier analysis of data from the 2005–2009 BRFSS, which reported increases in both blood cholesterol screening and elevated levels over time (6). From 2019 to 2023, the slight decrease in blood cholesterol screening during the preceding 5 years may reflect disruptions in routine health care due to the COVID-19 pandemic, which resulted in fewer in-person health care visits (9,10). Studies have documented a decline in the use of preventive care during the pandemic, including cholesterol screening, which was likely attributed to factors such as social distancing protocols, clinic closures, and patient hesitancy to seek in-person care (9,10). A single-center study reported that cholesterol testing rates were 39% lower during the first wave of the pandemic in 2020 compared with the same period in 2019 (9). Another analysis based on a nationwide web-based survey noted that more than 40% of adults delayed or avoided medical care during the pandemic (10). These changes, driven by shifts in public health priorities and limited access to care, may have contributed to delays in the detection and management of high blood cholesterol. In addition, the seemingly contrasting trends in screening prevalence over the past 5 years versus the past 1 year may be influenced by pandemic-related disruptions and recovery dynamics. The slight decline in 5-year screening may result from missed screenings during the early stage of the pandemic (2020–2021). While the modest uptick in screening within the past year could indicate a postpandemic rebound effect, with people likely resuming preventive services in 2023 after experiencing delays caused by the pandemic. This divergence between short-term and long-term screening prevalence underscores the importance of monitoring time-specific trends.

In this study, we observed an increase in the prevalence of self-reported high blood cholesterol from 2019 to 2023. Data from the 2017–2023 NHANES demonstrated that the prevalence of high total cholesterol (≥240 mg/dL) was higher during 2021–2023 compared with 2017–2020, although this change was not statistically significant (11). Studies based on earlier NHANES data have shown improvements in lipid measurements among adults over the past decades (12,13). Gao and colleagues analyzed data from the 1999–2018 NHANES and reported significant reductions in mean total cholesterol, triglycerides, and LDL-C levels among adults, particularly between 1999 and 2012 (12). Similarly, Aggarwal et al found improved lipid concentrations and greater lipid control from 2007–2008 through 2017–2018 (13). However, recent significant health-impacting events, such as the COVID-19 pandemic, may have disrupted this progress. For instance, lifestyle changes and mental health issues associated with the pandemic likely contributed to the rise in the prevalence of high blood cholesterol levels (14), as reduced physical activity and elevated stress and anxiety levels are known risk factors (15,16).

Furthermore, given that the BRFSS cholesterol data are based on self-reported responses, the observed increase in high blood cholesterol may also reflect improvements in diagnostic practices and patient–provider communication, rather than solely a rise in population cholesterol levels. Over the past decade, clinical management has shifted from a threshold-based approach (eg, optimal LDL-C levels <100 mg/dL) to a risk-based strategy that identifies populations most likely to benefit from targeted therapy (3,17). This shift in treatment guidelines has also influenced diagnostic practices by promoting the use of multiple clinical factors for risk assessment. The *2018 ACC/AHA Guideline on the Management of Blood Cholesterol* and the *2019 ACC/AHA Guideline on the Primary Prevention of Cardiovascular Disease* further expanded the scope of risk assessment by incorporating several key elements beyond traditional risk factors (3,4). This included risk-enhancing factors such as a family history of premature ASCVD, chronic inflammatory conditions, elevated lipid biomarkers, and other comorbidities (3,4). These updates may have prompted clinicians to broaden their screening practices, facilitating earlier detection of high blood cholesterol among at-risk people. As a result, the integration of risk calculation and shared decision-making tools likely enhanced provider recognition and patient notification, contributing to the increased reporting of elevated cholesterol levels over time.

Although women had a slightly greater relative increase in self-reported high blood cholesterol from 2019 to 2023, men continued to report higher prevalence in 2023, indicating higher overall awareness in men. Research has demonstrated that clinicians may underestimate women’s cardiovascular risk or attribute their symptoms to other causes, leading to delayed diagnosis and undertreatment of lipid disorders (18). Additionally, Cushman et al reported a significant decline from 2009 to 2019 in women’s awareness that heart disease is their leading cause of death, signaling persistent gaps in public health messaging and risk perception (19). These findings emphasize the importance of sex-specific strategies, such as improving risk communication, enhancing follow-up care, and increasing community outreach to optimize cholesterol management for both men and women.

Substantial state-level variations exist in the prevalence of screening for and awareness of high blood cholesterol. Our results indicate that cholesterol screening prevalence was generally lower in Mountain West states in 2023, which is consistent with observations of reduced use of preventive care services in sparsely populated areas, and a lower density of primary care providers in certain states within this region (20,21). From 2019 to 2023, we identified 34 states that experienced a significant rise in the prevalence of self-reported high blood cholesterol. These findings may reflect variations in public health infrastructure, insurance coverage, socioeconomic status, and access to care at the state level (22,23). Additionally, we found that Appalachian and Southern states reported the highest prevalence of high blood cholesterol in 2023, which aligns with the literature highlighting the significant burden of cardiovascular disease risk factors and associated mortality rates in these regions (24–26). The geographic differences underscore the importance of disaggregated surveillance and geographically tailored interventions. By recognizing state-specific trends in screening and awareness for elevated blood cholesterol, public health professionals and policymakers can prioritize areas most affected, tailor health communication strategies, and allocate resources more effectively.

Preventing high blood cholesterol involves adopting a heart-healthy diet, engaging in regular physical activity, and maintaining a healthy lifestyle. Reducing the intake of foods high in saturated fat, trans fat, and sodium, while increasing the consumption of fiber-rich foods such as fruits, vegetables, and whole grains, can contribute to lower cholesterol levels (27). It is also recommended to engage in at least 150 minutes of moderate-intensity aerobic exercise each week to maintain a healthy weight (27).

This report is subject to several limitations. First, it relies on self-reported information, which may be subject to recall bias and could underestimate or overestimate the true prevalence of high blood cholesterol. Second, although self-reported cholesterol data provide valuable insights into cholesterol awareness, they do not specify lipid panel components such as total cholesterol or LDL-C values, which may limit the assessment of cardiovascular risk profiles. Third, adults aged 18 to 20 years were included, in line with prior surveillance (6). Although guidelines recommend screening for lipid disorders starting at age 20, and there is insufficient evidence to support general screening in those aged 18 to 20 years, some people may benefit based on specific risk factors (3,4). Finally, the COVID pandemic overlapped with 2 of the study years (ie, 2021 and 2023), which likely influenced preventive health behaviors. However, this analysis was not structured as an interrupted time series or pre–post design. Additional research could consider incorporating earlier cycles to evaluate pandemic-specific effects on cholesterol screening and awareness.

This report highlights the population-level differences in screening and awareness for high blood cholesterol. The findings can guide national programs, such as the US Department of Health and Human Services’ *Million Hearts 2027* national initiative that aims to prevent one million heart attacks and strokes in 5 years (28), in developing evidence-based tools and resources to improve cholesterol management. To achieve the *Healthy People 2030* goal of reducing blood cholesterol (29), interventions and resources focused on enhancing awareness about the importance of cholesterol management should be prioritized (30). In addition, efforts such as regional campaigns, local partnerships, and integration with community health systems should be tailored to address the specific needs and challenges of each state’s population.
